# Primary Pancreatic Lymphoma in a Patient with Maturity Onset Diabetes of the Young Type 3

**DOI:** 10.4084/MJHID.2012.005

**Published:** 2012-01-18

**Authors:** Valentina Bozzoli, Maria Chiara Tisi, Luigi Pianese, Stefano Tumini, Vittoria Rufini, Maria Lucia Calcagni, Dario Pitocco, Alberto Larghi, Luigi Maria Larocca, Giuseppina Massini, Luciana Teofili, Francesco D’Alò, Stefan Hohaus

**Affiliations:** 1 Istituto di Ematologia, Università Cattolica del Sacro Cuore, Roma, Italy; 2 U.O. di Laboratorio di Analisi Cliniche e Microbiologiche, Settore Medicina Molecolare Azienda Sanitaria Unica Regionale ASUR n°13 Ascoli Piceno, Italy; 3 Clinica Pediatrica, Servizio Regionale di Diabetologia Pediatrica, Università di Chieti, Italy; 4 Istituto di Medicina Nucleare, Università Cattolica del Sacro Cuore, Roma, Italy; 5 Istituto di Medicina Interna, Università Cattolica del Sacro Cuore, Roma, Italy; 6 U.O. Endoscopia Digestiva Chirurgica, Università Cattolica del Sacro Cuore, Roma, Italy; 7 Istituto di Anatomia Patologica, Università Cattolica del Sacro Cuore, Roma, Italy

## Abstract

Primary pancreatic lymphoma (PPL) is an extremely rare disease which occurs in pancreas, accounts for less than 1% of extra-nodal malignant lymphomas and 0,5% of cases of pancreatic masses. We report the case of PPL in a 15 year-old boy suffering from Maturity Onset Diabetes of the Young type 3 (MODY3) diagnosed at the age of 1 year.

## Introduction

Diabetes Mellitus has been reported as presenting symptom associated to Primary Pancreatic Lymphoma (PPL), related to the lymphoma infiltration of pancreatic tissue and the following exocrine and endocrine insufficiency. Nevertheless, the onset of isolated PPL in a patient suffering from a monogenic type of diabetes has not been previously reported. Here we describe the case of a 15 years old young man with a pediatric diagnosis of Type I Diabetes Mellitus made at 1 year of age and later redefined as Maturity onset Diabetes of the young type 3 (MODY3) according to immunologic and molecular assays, who developed a PPL, a rare form of extranodal lymphoma more commonly diagnosed in advanced age.^1^ MODY3, a monogenic form of non-insulin-dependent diabetes, related to heterozygous germline mutation in HNF1A, has been considered a precancerous condition, predisposing the different type of tumor. We think that the uncommon association of these two rare diseases is worth being reported.

## Case Report

A 15-year old boy with a diagnosis of Diabetes Mellitus made from the age of 1 year, was admitted to the hospital reporting one-month history of jaundice. He presented in good general conditions, without any abdominal pain or B symptoms. Physical examination revealed skin and scleral jaundice, no organomegaly or peripheral lymphoadenopathy.

Blood chemical assays showed abnormal liver function tests (GOT 73 UI/l, GPT 144 UI/l, serum alkaline phosphatase 583 UI/l, total bilirubin 10,61 mg/dl), elevated LDH (504 UI/l) and beta2-microglobulin (3,2 mg/l). WBC count and platelets count were within the normal range, whereas a normochromic normocytic anemia was detected (hemoglobin 10.6 g/dl). Serologic tests for HIV, HBV, HCV, CMV, EBV and Toxoplasma were negative. Tumor markers included CA19-9, alpha-Fetoprotein and CEA were within normal ranges.

The CT abdominal scan showed a 4.0×3.2 cm low density tumor in the head of the pancreas, heterogeneously enhancing and obstructing the common bile duct and the main pancreatic duct, many centimetric peripancreatic lymphadenopathies and normal spleen and liver. The patient underwent Endoscopic Retrograde Cholangiopancreatography (ERCP) and a stent was placed in the common bile duct, obtaining the improvement of the jaundice. An endoscopic ultrasound (EUS) guided fine needle aspiration biopsy (FNAB) was performed and cytological examination revealed a massive infiltration by CD20 and LCA expressing lymphocytes.

**Figure 1:**
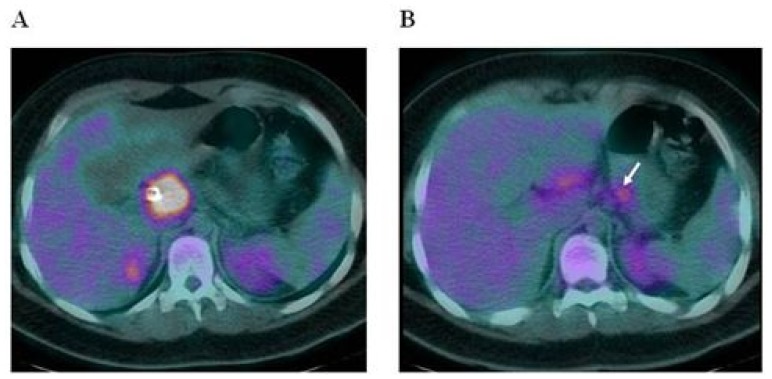
^18^F-FDG PET/CT showed increased glucose metabolism in a larger lesion in the head of pancreas (Figure 1A), as well as in one more smaller area in the tail of the pancreas (pointed by the arrow) and in one lymph node in the hepato-gastric ligament (Figure 1B).

The patient was then admitted to our Department of Hematology where he completed a CT scan of the head, neck and chest, showing no significant lymphadenopathies or parenchymal lesions. Bone marrow aspiration and trephine biopsy revealed no lymphoma involvement. Fluorine-18-Fluoro-deoxy-glucose positron emission tomography/computed tomography (^18^F-FDG PET/CT) was performed using a hybrid scanner (Philips Gemini GXL) and showed increased glucose metabolism in the known lesion in the head of pancreas (Figure 1A), as well as in one more smaller area in the tail of pancreas and in one lymph node in the hepato-gastric ligament (Figure 1B).

**Figure 2:**
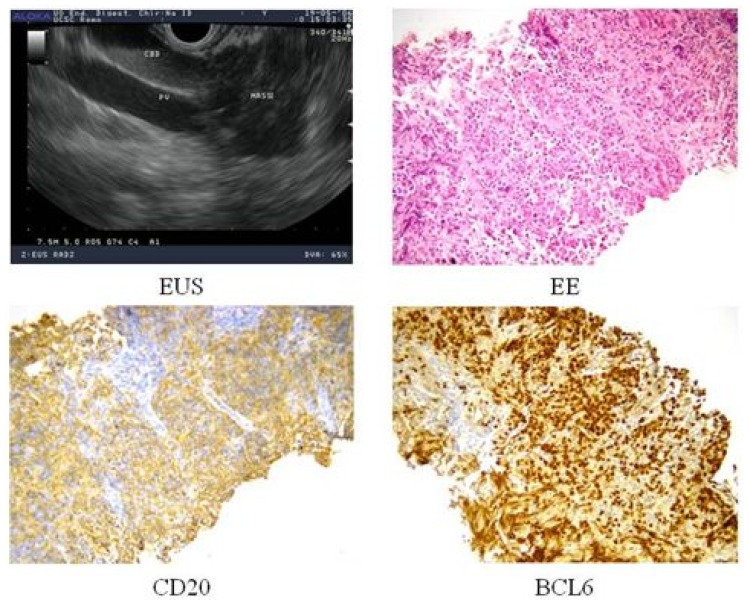
A) Endoscopic ultrasound (EUS) of the pancreatic lesion; B) hematoxylin and eosin staining of pancreatic bioptic specimen; C) CD20 immunostaining; D) BCL-6 immunostaining.

To have a more detailed histopathological diagnosis, an EUS-guided Trucut biopsy was performed and the pathology report documented the complete substitution of pancreatic parenchyma by a large blasts proliferation, expressing CD20, PAX-5, CD10, BCL-6 and MIB-1 proliferative index of 70%, suggestive of Diffuse Large B Cell Lymphoma (DLBCL) of the pancreas (Figure 2). Considering the extensive and multifocal pancreatic involvement associated with the involvement of peripancreatic lymph nodes, the final diagnosis was primary pancreatic DLBCL, stage IV_EA_, age-adjusted International Prognostic Index (I.P.I.) 2 (stage IV, elevated LDH).

Patient was treated with the immunochemotherapy schedule R-CHOP-14 (Rituximab 375 mg/mq, Cyclophoshamide 750 mg/mq, Doxorubicin 50 mg/mq and Vincristin 2 mg on day 1, Prednisone 100 mg/die from day 1 to day 5, Filgrastim from day 9 to 12, repeated every 14 days), with the addition of intrathecal prophylaxis with Methotrexate at each cycle. Blood chemical tests performed after 4 courses showed a normalization of bilirubin levels (total bilirubin 0.6 mg/dl) and the CT scan revealed a volumetric reduction of the larger pancreatic mass (3.5 × 2.9 cm). Considering the partial response, the treatment was intensified with a 3 courses multiagent immunochemotherapy Ri-MiCMA (Rituximab 375 mg/mq on day 1, Mitoxantrone 10 mg/mq on day 1, Carboplatin 100 mg/mq from day 1 to day 4, Cytarabine 2 g/mq on day 5, Methylprednisolone 750 mg from day 1 to 5, repeated every 21 – 28 days), with collection of peripheral CD34+ stem cell and followed by autologous stem cell transplantation. The achievement of a complete remission was documented by a ^18^F-FDG PET/CT scan performed at the end of the treatment. After almost 5 years of follow-up, the patient is in complete remission and in good general condition.

## Discussion

PPL is an extremely rare disease occurring in pancreas, with or without the involvement of peripancreatic lymph nodes, and accounts for less than 1% of extra-nodal malignant lymphomas and 0,5% of cases of pancreatic masses.^1^ Diagnostic criteria by Dawson et al.^2^ include: 1- neither superficial lymphoadenopathy nor enlargement of mediastinal lymph nodes; 2- a normal leukocyte count in peripheral blood; 3- main mass in the pancreas with lymph-nodal involvement confined to the peripancreatic region; 4-no hepatic or splenic involvement. The clinical manifestations and imaging results of PPL resemble other pancreatic cancers, such as adenocarcinoma, but unlike this, PPL are potentially treatable even if diagnosed at an advanced stage. The presenting symptoms and signs include epigastric pain, weight loss and jaundice, while other systemic symptoms such as fever, chills and night sweats are uncommon. PPL shows a strong male predominance (male to female ratio of 7:1), with median age of 55 years ranging between 35 and 75.^1^

So far less than two hundred cases have been described in literature consisting mainly in case reports or small case series.^3^ The etiology is unknown and no association have been described with common viral agents involved in the development of malignant lymphoma. Familial cases of PPL have been described.^4^

Diabetes Mellitus has been reported as presenting symptom associated to PPL, related to the lymphoma infiltration of pancreatic tissue and the following exocrine and endocrine insufficiency, with spontaneous recovery of pancreatic function after treatment-induced lymphoma remission.^5^,^6^

This patient suffered from Diabetes Mellitus from 1 year of age, which was initially defined as Type 1 Diabetes Mellitus, because it was the most likely diagnosis according to age and presentation almost 2 decades ago. Following the diagnosis of PPL, considering the intriguing hypothesis of association between extranodal lymphoma and organ-target autoimmune disease, as observed for other types of lymphoma, the diagnosis of Diabetes Mellitus was revised according to immunological and genetic tests. Immunological assays including anti-islet-cell, anti-GAD65, and anti-insulin autoantibodies were negative. Since patient was obese, had constant insulin needs, and had a familiar history of diabetes mellitus and glucose intolerance, the diagnosis of MODY was taken in consideration. Notably, genetic analysis of glucokinase (GCK) and hepatocyte nuclear factor-1 alpha (HNF1A) genes, whose mutations are the most common cause of MODY accounting for approximately 70% of cases, was performed using a previously described procedure.^7^ Direct sequencing of HNF1A gene resulted in the identification of a missense mutation p.G31D in exon 1 caused by a G>A transition at nucleotide 92 resulting in the substitution of glycine with aspartic acid at codon 31 allowing the redefinition of the initial diagnosis as MODY3.^8^

MODY refers to any of several hereditary forms of diabetes that are defined at the molecular genetics level by mutations in different genes accounting for an estimated 1–2% of diabetes in Europe. These monogenic forms are characterized by early onset (usually before 25 years of age and frequently in childhood or adolescence), autosomal dominant mode of inheritance and a primary defect in pancreatic beta cell function.^9^ Heterozygous mutations of HNF1A gene are associated with MODY3.^10^ HNF1A is a transcription factor that is thought to control a regulatory network important for differentiation of beta cells. Mutations of this gene lead to reduced beta cell mass or impaired function. In experimental models, HNF1A deficiency led to impaired large-T-antigen-induced growth and oncogenesis in beta cells and enhanced proliferation in hepatocytes.^11^

An association between a benign proliferation (such as liver adenomyomatosis) and MODY3 was reported, probably based on the involvement of a biallelic inactivation of HNF1A in hepatic adenomas occuring either by double somatic events or in association with a germ line HNF1A mutation. These results have stressed that HNF1A associated with MODY3 met the genetic criteria of a classical tumor suppressor gene.^12^

Diabetes Mellitus has been associated to a moderately increased risk of non-Hodgkin lymphoma as well as other malignancies, including endometrial, ovarian, breast, gallbladder, liver, colon and pancreatic cancer, probably related an altered immune function and a relatively immunodeficient state observed in diabetic patients.^13^ No reports have been so far published about the involvement of HNF1A in lymphomagenesis. Unfortunately we had not enough bioptic material to study acquired double-hit mutations of HNF1A on pancreatic lymphoma. Nevertheless, since both conditions of MODY3 and PPL are quite uncommon, in particular considering the young age of the patient, their interesting association could not be casual but be related to a possible link involving HNF1A mutations, primary pancreatic beta cell dysfunction, diabetes mellitus and lymphoma development. We thought that this case is worth being reported and further investigation of this issue are warranted.
